# Selective Small Molecule Stat3 Inhibitor Reduces Breast Cancer Tumor-Initiating Cells and Improves Recurrence Free Survival in a Human-Xenograft Model

**DOI:** 10.1371/journal.pone.0030207

**Published:** 2012-08-06

**Authors:** Bhuvanesh Dave, Melissa D. Landis, Lacey E. Dobrolecki, Meng-Fen Wu, Xiaomei Zhang, Thomas F. Westbrook, Susan G. Hilsenbeck, Dan Liu, Michael T. Lewis, David J. Tweardy, Jenny C. Chang

**Affiliations:** 1 The Methodist Cancer Center, Houston, Texas, United States of America; 2 Department of Infectious Diseases, Departments of Molecular & Cellular Biology and Radiology, Baylor College of Medicine, Houston, Texas, United States of America; 3 Department of Biochemistry and Molecular Biology, Departments of Molecular & Cellular Biology and Radiology, Baylor College of Medicine, Houston, Texas, United States of America; 4 Lester and Sue Smith Breast Center, Departments of Molecular & Cellular Biology and Radiology, Baylor College of Medicine, Houston, Texas, United States of America; Penn State Hershey Cancer Institute, United States of America

## Abstract

Metastasis and disease relapse are hypothesized to result from tumor initiating cells (TICs). Previously, we have defined a CD44+/CD24−/low mammosphere-forming tumorigenic 493-gene signature in breast cancer. Stat3 was identified as a critical node in self-renewal based on an ongoing lentiviral shRNA screen being conducted in two breast cancer cell lines SUM159 and BT549. In corroborating work, targeting the SH2 domain of Stat3 with a novel small molecule decreased the percentage of cells expressing TIC markers (CD44+/CD24−/low and ALDH+) and mammosphere formation in p-Stat3 overexpressing human breast cancer xenografts in SCID-beige mice. Importantly, we observed a four-fold improvement in the 30-day recurrence-free survival relative to docetaxel alone with the addition of the Stat3 inhibitor in the chemoresistant tumor model. Thus, these findings provide a strong impetus for the development of selective Stat3 inhibitors in order to improve survival in patients with p-Stat3 overexpressing tumors.

## Introduction

Despite significant advances in breast cancer biology and a multitude of clinical trials, progress in the treatment of advanced breast cancer, has been limited. Clinical trials to date have been based on a model of carcinogenesis best described as random or “stochastic” in which all cells within a tumor are considered more-or-less equally malignant. The tumor-initiating cell (TIC) hypothesis is a fundamentally different model in which it is proposed that a sub-population of cells retains key stem cell properties, including self-renewal, which initiates and drives tumor formation.

Recent research by our group and others has provided strong support for the TIC model, which provides a mechanism for resistance to conventional treatment, as well as an explanation for relapse and metastases. Our group had shown that TICs are intrinsically therapy resistant; residual tumors after chemotherapy are enriched for tumorigenic CD44^+^/CD24^−/low^ cells [1], [2], which show enhanced mammosphere-forming efficiency (MSFE) [3] and display accelerated outgrowth in xenograft transplants in immunocompromised SCID/Beige mice [4]. In addition, we identified a tumorigenic signature of 493 differentially expressed genes comprising the overlap of two enriched tumor-initiating cell populations (CD44^+^/CD24^−/low^ vs. bulk tumor cells and mammospheres vs. primary tumor) using biopsies obtained from women with primary breast cancer [2].

The main goals of this paper are: 1) to identify key regulatory pathways responsible for self-renewal based on ingenuity analysis of the 493 gene tumorigenic signature and an ongoing shRNA knowdown screen of this signature and the effects on mammosphere forming efficiency (MSFE), a surrogate in vitro assay for stem cell self-renewal, in two triple negative claudin-low like tumor cell lines (SUM159 and BT549) (Asterand Inc, MI, USA and ATCC, Maryland USA respectively) that have increased expression of many genes present in the TIC signature based on microarray analysis of these cell lines (data not shown), and 2) to examine the effect of targeting one of the identified pathways using a novel small molecule Stat3 inhibitor in two human cancer in mouse xenograft models, which have been well characterized and shown to mimic triple negative human breast cancer. Stat3 (Signal transducer and activator of transcription 3) is an oncogene activated in many cancers including breast, prostate, lung, head and neck and colon, liver, pancreas, and multiple myeloma [5], [6], [7]. Using the structure of the phosphotyrosyl peptide binding pocket within the Stat3 Src homology (SH) 2 domain in a virtual ligand screen, we recently identified a small molecule inhibitor, C188 that blocks two steps in Stat3 activation–receptor recruitment and homodimerization [8]. C188 was tested *in vivo* in two different triple negative breast cancer human xenograft models in SCID beige mice to determine the effects of Stat3 inhibition alone and in combination with chemotherapy. These “human-cancer-in-mouse” breast cancer xenograft model systems were derived directly from primary patient tumors into immunocompromised mice, and thus results obtained from these models may be translated to human disease. Our results demonstrate that C188 reduced tumor volume in combination with chemotherapy, decreased TICs resulting in reduction in tumor recurrence rates when compared to chemotherapy alone.

**Figure 1:**
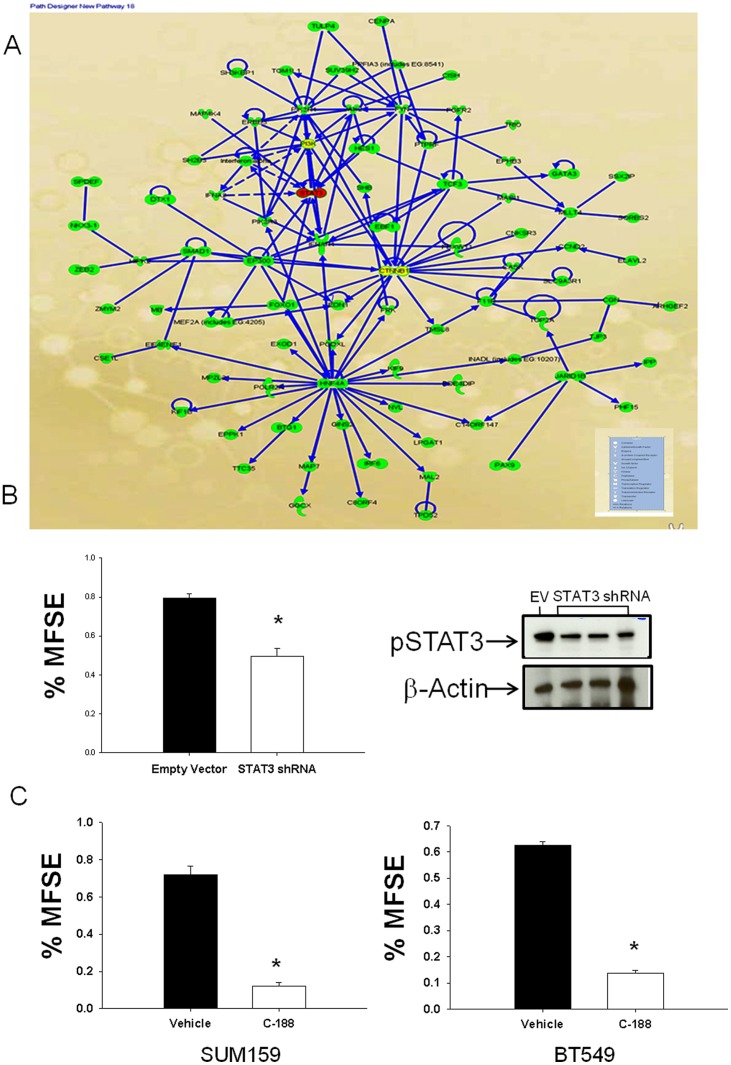
Ingenuity Analysis identifies Stat3 as an important target for TIC self renewal. (A) Ingenuity Analysis of 493-gene tumorigenic stem cell signature, looking for direct and indirect interactions of these genes among themselves identified Stat3 as one of the important targets of TIC self renewal. (B) Mammosphere forming efficiency in SUM159 treated with Stat3 shRNA vs. empty vector (EV) showed a significant decrease (p<0.05). Data depicted as Mean+ SEM. Western analysis of empty vector vs. Stat3 shRNAs treated cells depicts reduction in pStat3 levels upon treatment. (C)Mammosphere forming efficiency for SUM 159 and BT 549 cells treated with C188 at 10 µM concentration. Significant decrease in mammosphere formation upon treatment with C188 (p<0.05). Data depicted as Mean+SEM.

## Results

### Ingenuity analysis identifies Stat3 as an important target in TIC self renewal

Ingenuity analysis of the 493-gene tumorigenic gene signature was performed to identify the key nodes and players involved in TIC self renewal. Along with other factors, Stat3 was identified as an important target for TICs (Figure 1A). Further, in ongoing screen of lentivirally expressed shRNAs (pGIPZ-shRNAmir library, Open Biosystems) to disrupt function of candidate regulators of tumor-initiating cells, we identified shRNAs targeting Stat3 decreased mammosphere formation efficacy (MSFE). The shRNAs were tested in two claudin-low cell lines (SUM159 and BT549) using a high throughput 96-well MS formation assay (Figure S1 and Procedures S1). Additionally, we tested three lentivirally targeted Stat3 shRNAs in SUM159 (Fig. 1B) and found that these shRNAs decreased pStat3 levels in 72 hrs. This was associated with a corresponding decrease in MSFE in 4 days. A selective Stat3 inhibitor C188 was tested for effect on MSFE on SUM159 and BT549 cell lines. A significant decrease in MFSE was observed 4 days after treatment, with C188 in both triple negative breast cancer cell lines (Fig. 1C).

### Effect of Stat3 inhibition and chemotherapy on tumor volume and the TIC fraction in chemoresistant BCM2665 xenograft model

**Figure 2:**
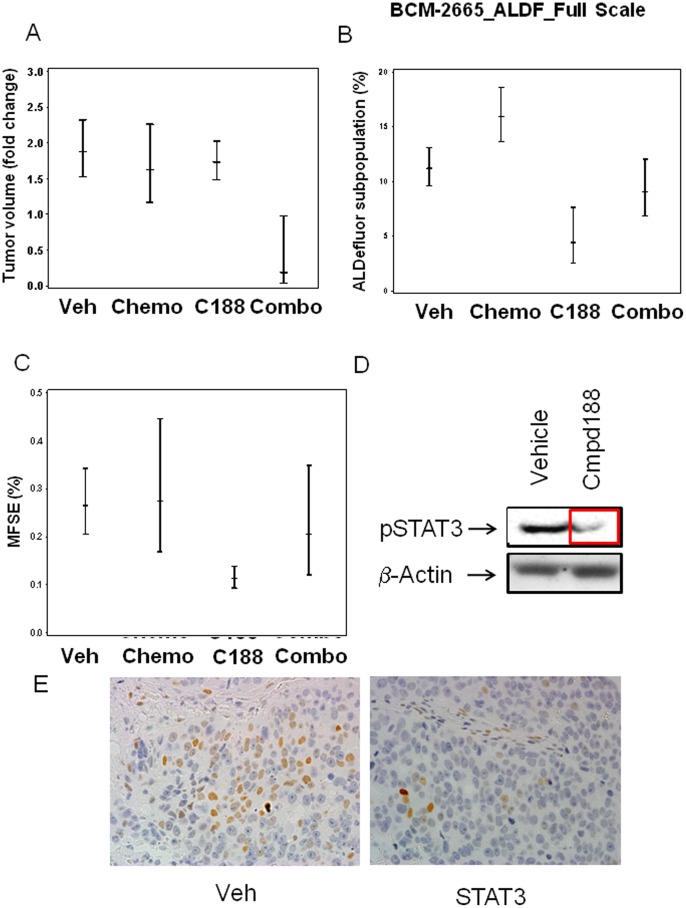
Chemoresistant BCM2665 transplanted in the fat pad of SCID Beige mice responds to combination of Stat3 inhibitor+docetaxel treatment, with decrease in tumor volume and TIC markers by FACS and MSFE. (A) Tumor volume fold change over time + SEM in 4 treatment arms of the chemoresistant BCM2665 model for the treatment period of 14 days. Significant decrease in mean tumor volume was noted between vehicle and combination of C188+docetaxel, and between chemotherapy and combination treatment (p<0.05). (B) FACS analysis of 10,000 cells BCM2665 tumor cells for ALDF+. Statistically significant decreases were observed with C188 vs. control (p<0.05), C188 vs. chemotherapy (p<0.05), and combination of C188+chemotherapy vs. chemotherapy alone (p<0.05). Data depicted as Mean + SEM. (C) Mammosphere forming efficiency of tumor cells harvested and seeded at 20,000 cells/well. Statistically significant decrease with C188 treatment vs. control (p<0.05), and C188 treatment vs. chemotherapy was observed (p<0.05). (D) The epithelial compartment of the BCM2665 tumors treated with C188 or vehicle control was isolated using laser capture microscopy. pStat3 levels were determined using Western blot analysis (representative picture shown). (E) Immunohistochemical analysis of pStat3Stat expression in Vehicle vs C188Stat treated BCM2665 tumor tissue (representative picture shown).

Female SCID Beige mice (36 mice, 9 mice per treatment arm) were transplanted with small fragments (∼1 mm^3^) of BCM2665 tumors, which were allowed to grow to ∼100–1000 mm^3^. Resulting tumor-bearing mice were then randomized into the four arms, namely vehicle, docetaxel (20mg/kg), Stat3 inhibitor (C188) (12.5 mg/kg), and the inhibitor+docetaxel combination. Tumor volume was monitored twice weekly using caliper measurements. Neither the Stat3 inhibitor nor docetaxel as single agents showed any reduction in tumor volume, relative to vehicle treated controls (Fig. 2A). However, the combination group showed a statistically significant decrease in tumor volume as compared to vehicle or chemotherapy.

In MSFE assays (Procedures S1), which provide a measure of the proportion of cells capable of anchorage-independent growth, C188 showed a statistically significant decrease in MSFE relative to vehicle (p<0.05) (Fig. 2B) (Table S1). This effect was not observed in combination with docetaxel. FACS analysis (Procedures S1) of BCM2665 tumor cells (∼10,000 cells) demonstrated that the Stat3 inhibitor C188 reduced ALDH1 levels in treated samples. Specifically, when compared to the vehicle or chemotherapy groups, treatment with the Stat3 inhibitor C188 significantly reduced ALDH+ cells (p<0.05) (Table S2). In addition, as expected from our previously published results, chemotherapy treated samples showed a statistically significant increase in ALDH+ cells, consistent with the intrinsic chemoresistance of TICs (Dontu et al., 2005, [4] (p<0.05).

### Stat3 target engagement

Previous studies demonstrated that C188 inhibited Stat3 phosphorylation. To demonstrate target engagement using C188 by decrease in pStat3 levels, we performed laser capture microdissection (LCM) to isolate the epithelial compartment of the BCM2665 tumors treated with vehicle or C188 treated groups (Procedures S1). Western analysis was performed to determine the levels of pStat3 in the different treatment groups. These results demonstrate a reduction in the pStat3 levels in the C188 treated tumors vs. the vehicle treated group (Figure 2D).

### Effect of Stat3 inhibition in chemosensitive MC1 xenograft model

**Figure 3:**
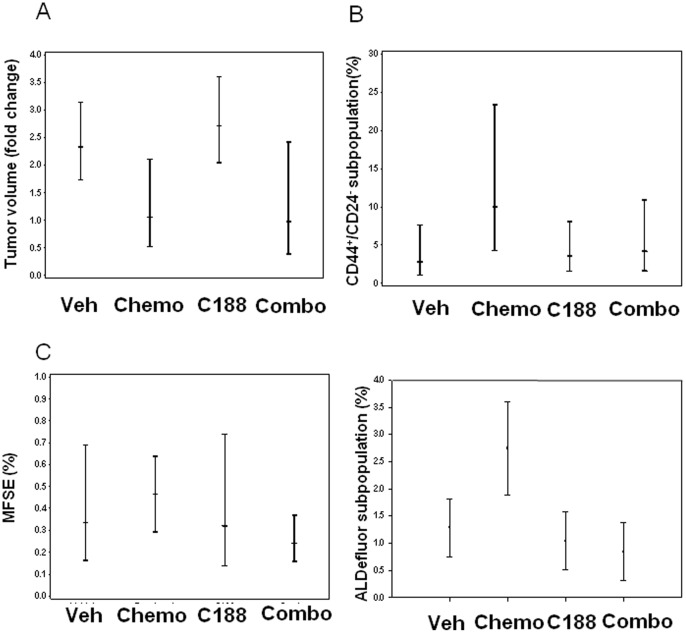
Chemosensitive MC1 transplanted in fat pad of SCID Beige mice, with non-significant decrease in TIC markers (ALDF, CD44+/CD24−, and MSFE) with Stat3 inhibitor compared to chemotherapy alone. (A) Tumor volume fold change over time (14 days). Decrease in mean tumor volume + SEM was observed with chemotherapy alone (chemosenstive tumor line). (B) FACS analysis of 100,000 cells MC1 tumor cells for CD44+/CD24−, ALDF. Data were depicted as Mean + SEM. Non-significant decrease in TIC markers (ALDF, CD44+/CD24) with Stat3 inhibitor compared to chemotherapy alone was observed. (C) Mammosphere forming efficiency of tumor cells seeded at 10,000 cells/ml. Data depicted as Mean + SEM. Non-significant decrease in MSFE with C188 compared to chemotherapy alone was observed.

As with BCM2665, female SCID Beige mice were transplanted with MC1 tumors, which were allowed to grow to ∼200–700 mm^3^, and then randomized into the same four treatment arms. C188 had no effect on tumor growth as a single agent. With chemotherapy, there was a significant reduction in tumor volume relative to the other treatment groups (Fig. 3A), while the combination C188 and docetaxel showed an intermediate growth curve between the two single agent arms (Fig. 3A).

In MSFE assays, there was no significant change as a consequence of any treatment. With FACS analysis, this decrease in tumor volume with chemotherapy was associated with a non-statistically significant increase in TICs as measured by CD44+/CD24−, ALDH+, and MSFE. In this chemosensitive xenograft model, with C188 alone, there was no change in TICs vs. vehicle control. However, the combination of C188+chemotherapy, there was a non-significant decrease in all TIC markers (CD44+/CD24−, ALDF+, and MSFE) when compared to chemotherapy alone (Fig. 3 C).

### Effect of combination of Stat3 inhibition and docetaxel on time to tumor recurrence (recurrence-free survival

**Figure 4:**
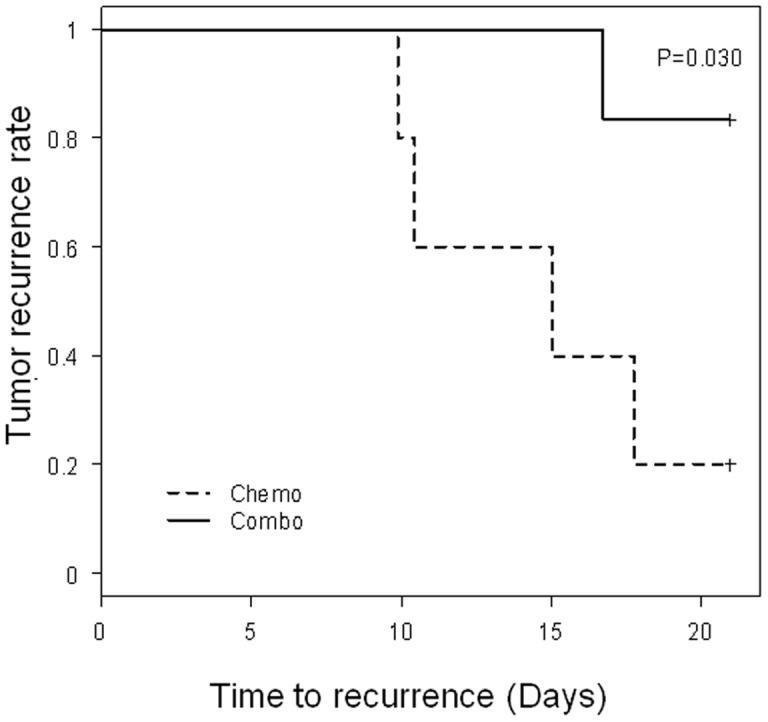
Kaplan Meier recurrence free survival with the combination with high dose docetaxel and C188 vs. chemotherapy alone. Female mice were transplanted in one mammary fat pad with chemoresistant breast cancer xenograft 2665A (ER/PgR/HER2-negative and p-Stat3-positive). After 6 weeks, mice with ∼200 mm^3^ tumors were randomized and treated with either 2 cycles of docetaxel (60 mg/kg) combined with daily dose of C188 (12.5 mg/kg; solid line) at the start of treatment vs. docetaxel alone (– line). Mice were observed daily after the end of all treatment (all the tumors had receded at that time). Time to tumor recurrence as denoted by tumors being measured to be larger than 50mm^3^ was significantly improved with the combination vs. chemotherapy alone (p = 0.030).

To evaluate the effect of Stat3 inhibition on recurrence, SCID Beige mice were transplanted with BCM2665 tumors and randomized to two groups (n = 6/group) at 6 weeks and treated with high dose docetaxel (60mg/kg) or the combination of high dose docetaxel and C188 for 14 days, per cycle, for two cycles. Complete tumor disappearance was observed in both arms at which time the treatment was stopped. The animals were then followed for tumor recurrence for a period of 20 days, and time to tumor recurrence was calculated using Kaplan Meier survival analysis. The time to recurrence was improved by ∼4-fold with the addition of Stat3 inhibitor to conventional chemotherapy compared to chemotherapy alone, with 30-day tumor recurrence rate of 20% (95% CI: 0.01–0.58) vs. 83% (95% CI: 0.27–0.97) (p = 0.03, Wilcoxon test) (Fig. 4), thus confirming that Stat3 inhibition decreased the TIC subpopulation and improved survival (Table S3).

## Discussion

We had previously demonstrated that the intrinsic resistance of TIC subpopulation to conventional chemotherapy, with an increase in CD44^+/hi^/CD24^−/low^/Lin^−^ cells, MSFE, and xenograft tumors on transplantation [4]. Others have also noted that tumors rich in CD44+ have a significantly worse clinical outcome [9]. Based on these observations, we derived a tumorigenic gene signature from human patient tumors using flow sorting for CD44^+/hi^/CD24^−/low^/Lin^−^ and mammosphere assays [2]. The goal with this study was to identify and begin to target the major pathways involved in the self-renewal of breast TICs. Previous studies targeting tumor initiating cells have had varying degrees of success [10], [11]. We identified key nodes of several pathways using ingenuity analysis including growth factor signaling pathway wnt signaling pathway and the Notch pathway and Stat3 pathway. Here, we confirm that Stat3 is a promising pathway target regulating tumor growth. Inhibiting this pathway led not only a decrease in cells expressing TIC markers but also to a significant delay in time to tumor recurrence in human-cancer-in-mouse xenograft models.

We tested C188 on the two cell lines *in vitro* (SUM159 and BT549) and demonstrated that C188 was indeed effective in reducing MSFE in cell lines (Fig. 1C). In order to study the effects of inhibiting Stat3 *in vivo*, we utilized human-cancer-in-mouse xenograft models, and confirmed that Stat3 inhibition not only reduced the frequency of cells expressing TIC markers as measured by flow cytometry (CD44+/CD24−, and ALDH) and MSFE, this inhibitor also significantly decreased time to tumor recurrence when compared to chemotherapy alone using a chemoresistant xenograft model.

Stat3 was originally termed acute-phase response factor (APRF) and was first identified as a transcription factor that bound to IL-6-response elements within the enhancer-promoter region of various acute-phase protein genes [12]. In addition to IL-6 receptors, other signaling pathways are linked to Stat3 activation which include receptors for other type I and type II cytokine receptors, receptor tyrosine kinases, G-protein-coupled receptors and Src kinases [12], [13]. Targeted disruption of the mouse *Stat3* gene leads to embryonic lethality at 6.5 days [14], thus indicating that Stat3 is essential for early embryonic development [15]. Interestingly, tissue-specific deletion of Stat3 in the hematopoietic lineage did not result in impaired blood cell production, suggesting that Stat3 inhibition may not exacerbate bone marrow suppression observed with chemotherapy agents.

Cytokines have been shown to play an important role in the function and modulation of tumor initiating cells. Stat3 mediates its activity in an IL-6 dependent manner [12], [13]. Previous studies have demonstrated that IL-8 dependent signaling and its receptor CXCR1 may be important in TIC signaling. [11]. Our data demonstrate that Stat3 signaling, is also important for TIC self renewal, and suggest that cytokine mediated signaling in TICs which needs to be further explored.

Constitutive Stat3 activity has been observed in ER-negative invasive breast cancer samples and metastatic cell lines. Stat3 activation within these cancer tissues and cells leads to an in increased level of anti-apoptotic proteins including Bcl-2 and Survivin [16], [17]. Stat3 activity contributes to oncogenesis in breast and other cancer systems, up-regulates anti-apoptotic proteins through several pathways, enhances cell proliferation, induces angiogenesis, and suppresses immune responses [18]. Stat3 has been postulated as a potential target against TICs for various tumor types, including glioblastoma multiforme [19], [20]. In addition, targeted disruption of Stat3 in the skin demonstrated the role of Stat3 and follicular stem cells in tumor initiation [21]. Thus, Stat3 is a potential high-yield target for drug development to treat several cancers including triple-negative breast cancers for which there are currently no currently approved molecularly targeted therapies.

In these experiments, we report in chemoresistant tumors that the combination of C188 plus chemotherapy decreased tumor volume after one cycle, and that the inhibitor alone resulted in a decrease in TICs as measured by standard flow cytometry markers and MSFE. The maximum effect of inhibiting TICs would be in maximally “debulked” disease, where the majority of the proliferating daughter cells are eliminated by conventional treatments. In the clinical scenario, this would represent adjuvant treatment, after completion of definitive therapy. With Stat3 inhibitor, the time to tumor recurrence was significantly improved by approximately 4-fold, when compared to chemotherapy alone. This result is highly encouraging and suggests that Stat3 inhibition may decrease time to relapse and improved outcomes in breast cancer patients.

In conclusion, inhibition of Stat3 by a novel, selective small molecule decreased the candidate TIC subpopulation in human cancers transplanted in mice, resulting in not only a decrease in tumor volume, but also prolongation of the time to tumor recurrence significantly. Future modifications of this small molecule, together with toxicity profile, would enable similar compounds to be tested clinically in breast and other cancers.

## Materials and Methods

### Stat3 shRNA knockdown

Lentiviral vectors encoding empty vector or three different Stat3 shRNAs in a pGIPZ vector (Open Biosystems Huntsville, AL) were used to transduce SUM159 and BT549 cells under low attachment conditions for 3–4 days. MFSE and western analysis was performed.

### Animals and Xenograft tumors

All animals were maintained in accordance with the NIH Guide for the Care and Use of Experimental Animals with approval from the Baylor College of Medicine Institutional Animal Care and Use Committee under protocol number AN2289 No approval was needed for the human IRB committee since the human cancer in -mouse xenografts are already established in mice.

### Stat3 Xenograft experiments

In vivo experiments were conducted study the effect of Stat3 inhibition using C188 (a small molecule that inhibits binding of the SH2 domain of Stat3 to its phosphotyrosyl peptide) [8] in two triple negative (estrogen receptor/progesterone receptor/HER2 negative) human cancer xenografts (BCM2665 and MC1) derived from primary human breast cancers, transplanted in the fat pad of SCID Beige mice (36 mice per xenograft line, 9 mice per treatment arm). When the tumors reached between 100–1000 mm^3^, mice were divided into four groups: (1) vehicle-treated (2) chemotherapy-treated (day 1 with one dose of docetaxel 20 mg/ml by intraperitoneal (i.p.) injection, (3) Stat3 inhibitor (day 1–14 with C 188 at 12.5 mg/ml (i.p.) (4) combination treatment with docetaxel (20mg/ml i.p. on day 1) and Stat3 inhibitor C188 (day 1–14 daily at 12.5 mg/kg i.p. Animals were sacrificed on day 14, 2 hrs after the final C188 treatment. The tumors were harvested and analysis for downstream effects of treatment performed.

To test our hypothesis that Stat3 inhibition by C188 will affect candidate TICs and decrease the time to relapse, we next examined its effect on time to recurrence *in vivo.* We conducted high dose studies since this xenograft model is resistant to docetaxel, where xenograft BCM2665 (with high levels of pStat3) were treated with high dose docetaxel (day 1 and day 15 at 60 mg/kg, i.p.) or the combination with high dose docetaxel (day 1 and day 15 at 60 mg/kg, i.p.) together with a daily dose of Stat3 inhibitor C188 (12.5 mg/kg, i.p.). After two cycles of therapy, disappearance of all tumors was observed in both groups. Treatments were then stopped, and the animals monitored for changes in body weights and time to tumor recurrence twice weekly.

### TIC analysis by FACS and mammosphere formation efficiency (MSFE)

The fraction of tumor-initiating cells in the xenograft tumors with the different treatment groups were processed by mincing the tumors and digesting them using collagenase type III, for 3 h at 37°C to dissociate the tumors into single cells. Changes in CD44+/CD24/Lin− and Aldefluor were analyzed, as previously described [4].

Mammosphere culture was performed as previously described [3], [4], [22]. Single cells were plated in ultra-low attachment plates (Corning, Acton, MA, USA). The cells were plated at a density of 40,000 viable cells/ml for BCM2665 in primary culture, and 10000 cells/ml in secondary culture, the amount was halved for MC1 tumor line, as these tumors had been shown previously to have higher mammosphere formation efficiency.

### Laser Capture Microdissection

Xenograft tumors were microdissected at 10x magnification with a power range of 60 mW–80 mW and pulse range of 2,500 µs–1100 µs using Veritas Microdissection Systems (Molecular Devices, Union City, CA). Protein was eluted from the laser capture microdissection (LCM) caps and used for western analysis for p-STAT3 pStat3 and β-actin.

### Statistical analysis

Statistical analysis and p values for tumor volume fold change, mammosphere formation efficiency and aldefluor assays are based on contrasts from a generalized linear model adjusted for multiple comparisons using Hommel method. For the tumor recurrence experiments, events are defined as first appearance of tumor >50 mm^3^ after completion of treatment. Time to recurrence was derived by Kaplan-Meier method, with differences compared using the generalized Wilcoxon test.

## Supporting Information

Figure S1 **shRNA screen for tumor initiating cells identifies Stat3 as an important node in the pathways.** Two triple negative breast cancer cell lines SUM159 and BT549, were infected with lentiviral shRNA's form open biosystems, targeting all the genes in our published tumorigenic signautre using high throughput mammosphere forming scree. This was followed by ingenuity analysis of the data to pictorially depict the pathways determined that Stat3 was an important component of the tumor initiating cell pathway.
(TIF)

Table S1 **Mammosphere forming efficiency Statistics.**
(XLS)

Table S2 **ALDH1 levels data statistics for BCM2665 tumor.**
(XLS)

Table S3 **Tumor volume fold change for MC1 tumor xenograft (statistics).**
(XLS)

Procedures S1 **Supplemental Experimental Procedures.**
(DOCX)
