# Correction: Selective Small Molecule Stat3 Inhibitor Reduces Breast Cancer Tumor-Initiating Cells and Improves Recurrence Free Survival in a Human-Xenograft Model

**DOI:** 10.1371/annotation/753d15c8-2321-4dc5-9b84-c47bd1bd1639

**Published:** 2012-09-28

**Authors:** Bhuvanesh Dave, Melissa D. Landis, Lacey E. Dobrolecki, Meng-Fen Wu, Xiaomei Zhang, Thomas F. Westbrook, Susan G. Hilsenbeck, Dan Liu, Michael T. Lewis, David J. Tweardy, Jenny C. Chang

There was an error in the author byline. The correct author list and affiliations are:

Bhuvanesh Dave^1^, Melissa D. Landis^1^, David J. Tweardy^2^, Jenny C. Chang^1^

1 The Methodist Cancer Center, Houston, Texas, United States of America,

2 Department of Infectious Diseases, Departments of Molecular & Cellular Biology and Radiology, Baylor College of Medicine, Houston, Texas, United States of America

