# Supplementary figures and images for: Selective Small Molecule Stat3 Inhibitor Reduces Breast Cancer Tumor-Initiating Cells and Improves Recurrence Free Survival in a Human-Xenograft Model
Source: PLoS One. 2012 Aug 6;7(8):e30207. doi: 10.1371/journal.pone.0030207 (PMC3412855; doi:10.1371/journal.pone.0030207)

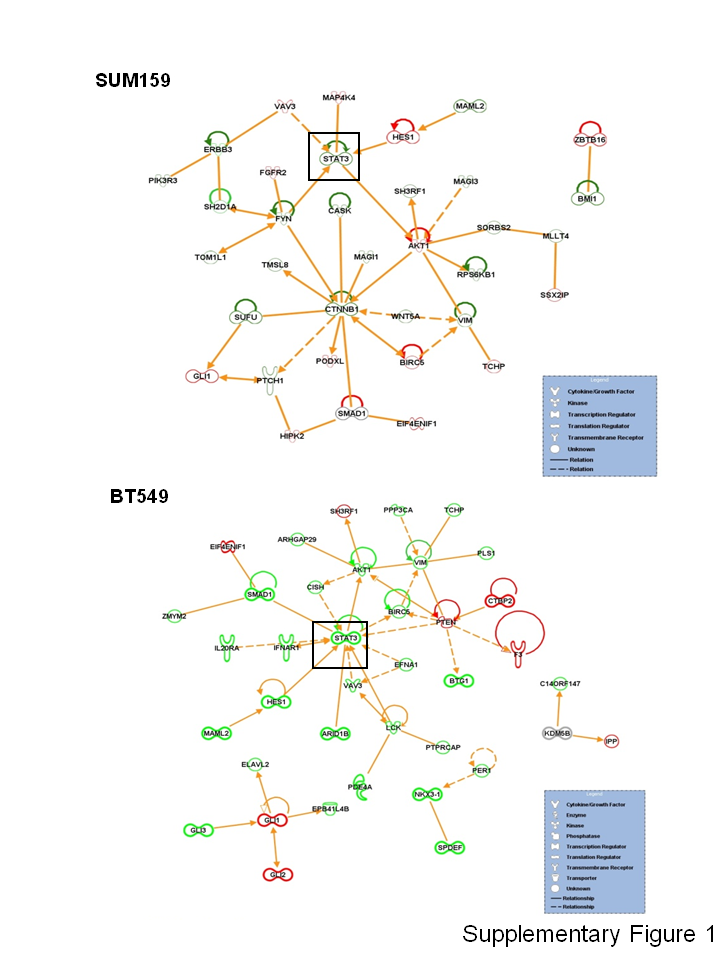

Supplement: Figure S1 — shRNA screen for tumor initiating cells identifies Stat3 as an important node in the pathways. Two triple negative breast cancer cell lines SUM159 and BT549, were infected with lentiviral shRNA's form open biosystems, targeting all the genes in our published tumorigenic signautre using high throughput mammosphere forming scree. This was followed by ingenuity analysis of the data to pictorially depict the pathways determined that Stat3 was an important component of the tumor initiating cell pathway. (TIF) [file pone.0030207.s001.tif]
