# Supplementary material for: Selective Small Molecule Stat3 Inhibitor Reduces Breast Cancer Tumor-Initiating Cells and Improves Recurrence Free Survival in a Human-Xenograft Model
Source: PLoS One. 2012 Aug 6;7(8):e30207. doi: 10.1371/journal.pone.0030207 (PMC3412855; doi:10.1371/journal.pone.0030207)
Supplement: Procedures S1 — Supplemental Experimental Procedures. (DOCX) [file pone.0030207.s005.docx]

*Supplemental Experimental Procedures:*

*FACS analysis*

The fraction of tumor-initiating cells in the xenograft tumors with the different treatment groups were processed by mincing the tumors and digesting them using collagenase type III, for 3h at 37^0^C to dissociate the tumors into single cells. Changes in CD44+/CD24/Lin- and Aldefluor were analyzed, as previously described ([Li et al., 2008](#_ENREF_14)). Briefly, cells were stained with primary antibodies anti-CD44 labeled APC (dilution 1:10, BD Biosciences), anti-CD24 labeled FITC (dilution 1:10, BD Biosciences), and H2KD PE was used to eliminate mouse cells and all lineage positive cells. Incubation was performed for 15 min on ice in HBSS (Hanks Balanced Salt Solution, GIBCO) with 2% FBS for the antibodies and 45 min for Aldefluor reagent. After incubation, cells were washed once with HBSS and were re-suspended in HBSS supplemented with 2% FBS. PI was added to the cells for live/dead gating prior to FACS analysis.

*Mammosphere formation assay*

For mammosphere culture, cells were grown in a serum-free mammary epithelial basal medium (MEGM) (Lonza Inc, Allendale 07401 NJ, USA) supplemented with B27 (Invitrogen, Carlsbad, CA, USA), 20 ng/mL EGF (BD Biosciences, San Jose, CA, USA), 1% heparin (Sigma Chemical Co., St. Louis, Missouri) in a humidified incubator (10% CO2: 95% air, 37̊ C for 14 days, as previously described ([Li et al., 2008](#_ENREF_16)). Mammosphere culture was performed as previously described ([Dontu et al., 2003](#_ENREF_7); [Dontu et al., 2005](#_ENREF_8); [Li et al., 2008](#_ENREF_16)). Single cells were plated in ultra-low attachment plates (Corning, Acton, MA, USA). The cells were plated at a density of 40,000 viable cells/ml for BCM-2665 in primary culture, and 10000 cells/ml in secondary culture, the amount was halved for MC1 tumor line, as these tumors had been shown previously to have higher mammosphere formation efficiency.

*Laser Capture Microdissection*

Xenograft tumors were sectioned into 5μM sections and transferred to membrane slides. The tissue slides were fixed as follows: 70% ethanol for 30 sec, 95 % ethanol for 1 min, 100% ethanol for 1 min, xylene for 5 min. The slides were removed from xylene solution and allowed to air dry for 5 min. The fixed slides were loaded into a Veritas laser capture microdissection (LCM) machine (Molecular Devices). The tissue samples were microdissected at 10x magnification with a power range of 60mW-80mW and pulse range of 2,500μs-1100μs using Veritas Microdissection Systems (Molecular Devices, Union City, CA). Protein was eluted from the laser capture microdissection (LCM) caps and used for Western analysis. The LCM proteins were quantified using BCA protein assay (Thermo-Fischer, Rockford IL). Equal amounts of proteins were loaded in each group and β-actin was used as a loading control. ECL reagent (Amersham Pharmacia) was used to develop the blots with pStat3 (#9145 Cell Signaling Technologies) and β-actin (#4970, Cell Signaling Technologies).

*Selective Stat3 shRNA knockdown*

We tested 3 different shRNAs against Stat3 from Open Biosystems (Huntsville, AL), which were encoded in the pGIPZ vector. These shRNAs were packaged into lentiviral particles using a second-generation lentiviral packaging system (Addgen,Cambridge, MA) in 293T cells plated on 96-well plates. The supernatant was then harvested for transduction and expression in a wide range of cell types. Specifically, lentiviruses were collected 3 days after transfection. This virus was used to transduce SUM159 cells under low attachment conditions and the cells were collected at 72 hrs (for Western analysis) or counted at 4 days for MSFE. The results were compared with negative control empty vector for analysis.
